# Researching the Impact of Service provider Education (RISE) Project — a multiphase mixed methods protocol to evaluate implementation acceptability and feasibility

**DOI:** 10.1186/s40814-022-01096-y

**Published:** 2022-07-02

**Authors:** Melissa Kimber, Meredith Vanstone, Gina Dimitropoulos, Delphine Collin-Vézina, Donna Stewart

**Affiliations:** 1 Offord Centre for Child Studies, Department of Psychiatry and Behavioural Neuroscience, McMaster University, BAHT 132, 1280 Main Street West, Hamilton, Ontario L8S 4K1 Canada; 2 Department of Health Research Methods, Evidence, and Impact, McMaster University, 1280 Main St West, Hamilton, ON Canada; 3 Department of Family Medicine, McMaster University, 1280 Main St West, Hamilton, ON Canada; 4 Faculty of Social Work, University of Calgary, MacKimmie Tower 413, 2500 University Dr NW, Calgary, AB Canada; 5 School of Social Work, McGill University, 3506 Rue University #300, Montréal, QC Canada; 6 Department of Pediatrics, McGill University, 1001 Decarie Blvd, Montréal, QC Canada; 7 Centre for Mental Health, University of Toronto and University Health Network, EN-7-229, 200 Elizabeth Street, Toronto, ON Canada

**Keywords:** Implementation science, Active Implementation Frameworks, Health professions’ education, Family violence, Mixed methods

## Abstract

**Background:**

Health and social service providers receive limited education on recognizing and responding to family violence. With adequate education, providers could be prepared to identify individuals subjected to family violence and help reduce the risk of associated impairment. Informed by the Active Implementation Frameworks, our research will determine the scope of strategies needed for the uptake and sustainability of educational interventions focused on family violence for providers. It will also determine the acceptability, feasibility, and proof-of-concept for a new educational intervention, called VEGA (Violence, Evidence, Guidance, Action), for developing and improving primary care provider knowledge and skills in family violence.

**Methods:**

This paper details the protocol for the *R*esearching the *I*mpact of *S*ervice provider *E*ducation (RISE) Project. The RISE Project follows a sequential multiphase mixed method research design; qualitative and quantitative data are being collected and integrated over three conceptually and methodologically linked research phases. Activities primarily occur in Ontario, Alberta, and Quebec. Phase 1 uses a sequential exploratory mixed method research design to characterize the scope and salience of learning and implementation needs and preferences for family violence education. Phase 2 will use an embedded mixed method research design to determine whether VEGA technology supports providers to achieve their family violence learning goals with effectiveness, efficiency, and satisfaction. Phase 3 will use a concurrent mixed method research design to determine acceptability, feasibility, and proof-of-concept for evaluating whether VEGA improves primary care providers’ knowledge and skills in family violence. This final phase will provide information on implementation strategies for family violence education in the “real world.” It will also generate data on provider recruitment, retention, and data completeness, as well as exploratory estimates of the effect for provider outcome measures proposed for a randomized controlled trial.

**Discussion:**

The RISE Project comprehensively integrates an implementation approach to improve family violence education for the health and social service professions. It will provide important information about factors that could influence the uptake and effectiveness of a health profession’s educational intervention into the real world, as well as provide foundational evidence concerning the tenability of using a randomized controlled trial to evaluate the impact of VEGA in primary care settings.

**Supplementary Information:**

The online version contains supplementary material available at 10.1186/s40814-022-01096-y.

## Background

Research consistently details the negative physical, emotional, and economic consequences of intimate partner violence (IPV), child maltreatment, and children’s exposure to IPV (collectively hereafter referred to as “family violence”) on the development and well-being of individuals, families, and communities [1–4]. Importantly, evidence indicates that exposure to one or more forms of family violence in childhood significantly increases an individual’s risk for further victimization over the life course [5–7]. Given the considerable overlap in the occurrence and health-related burdens associated with the various forms of family violence, health and social service providers (HSSPs), including physicians, social workers, nurses, midwives, as well as others, have been recognized as having a critical role in prevention and early intervention [8, 9]. However, several studies indicate that HSSPs have limited formal education related to family violence and that they experience challenges recognizing and responding to the various forms of family violence in their practice encounters [10–13]. In addition, evidence indicates an urgent need to increase the amount and quality of family violence education for HSSPs [11–15]; a scalable and efficacious educational intervention for HSSPs that addresses all forms of family violence has yet to be identified.

This paper details the protocol for the *R*esearching the *I*mpact of *S*ervice provider *E*ducation (RISE) Project. The RISE Project is a novel, multiphase mixed method research project. It is informed by the Active Implementation Frameworks (AIFs) and aims to determine the scope of strategies needed for the uptake and sustainability of educational interventions focused on family violence for HSSPs, as well as evaluate the uptake and educational impact of the Violence, Evidence, Guidance, Action (VEGA) educational intervention. VEGA (detailed below) is a publicly available, online educational intervention that was released in February 2020; it includes several pedagogical strategies to support increases in HSSP knowledge, attitudes, skills, and behaviors (KASB) related to recognizing and responding to all forms of family violence.

### Educational interventions for HSSP recognition and response to family violence

Five systematic reviews provide important information regarding existing educational interventions focused on family violence and which have been empirically evaluated [16–20]. This literature indicates that available interventions have tended to focus on physical/sexual IPV and have focused on physician, nurse, and dental professionals in the USA, UK, and Australia. Importantly, these interventions do not concord with recent evidence about best practices for safely recognizing and responding to family violence in clinical encounters. Several interventions have emphasized screening for family violence exposures, despite no evidence indicating that screening leads to measurable improvements in health outcomes among individuals exposed to family violence and in fact can place victims at greater risk of harm [21, 22].

Similarly, few of the educational interventions acknowledge the significant evidence detailing the complex overlap between IPV, children’s exposure to IPV, and other forms of child maltreatment [23–29]. Given the disproportionate impacts of IPV for women, the extent of overlap between IPV and child maltreatment, and that mandatory reporting laws have progressively recognized the harmful effects of children’s exposure to IPV, advocates have been clear about the need for educational programs to carefully consider responses to suspected and disclosed IPV in clinical practice.

Existing interventions have also focused on measuring changes to health professional attitudes and knowledge and have less often incorporated reliable assessments of change in practice skills and behaviors; for example, knowing that a client presentation meets the threshold for suspicion of child maltreatment and requires reporting to child protection authorities, referral to resources, or other types of intervention. One exception is the study by Pelletier and colleagues [30] which demonstrated significant improvements in reporting accuracy using vignette-based assessment methods, from pre- to post-education [30–32].

In sum, peer-reviewed empirical literature which evaluates the value and impact of educational interventions focused on family violence among HSSPs is disparate, does not consider all forms of family violence, does not reflect current evidence for best practices in recognition and response, and is inadequately designed for scalability across disciplines and contexts. We do not know which educational approaches reliably change HSSP knowledge and skill related to family violence.

Though not yet formally evaluated, the VEGA Family Violence Educational Resources (see: https://vegaproject.mcmaster.ca/) offer important potential to address the critical education gaps related to family violence among HSSPs. VEGA is a free, brief, and evidence-informed health professions educational intervention that aims to improve HSSP KASB related to recognizing and responding to family violence in clinical practice. Informed by the AIF, the RISE Project (outlined below) is the first formal evaluation of VEGA. The RISE Project is using a robust, multiphased mixed method research design that will (1) identify and develop implementation and evaluation supports for the pan-Canadian dissemination and implementation of family violence education, including VEGA, to HSSPs; (2) use user-testing methodology to determine if the technology of VEGA allows its users to achieve their learning goals effectively, efficiently, and satisfactorily in a self-directed learning format; and (3) determine acceptability, feasibility, and exploratory estimates of impact for implementing and evaluating VEGA as a health professions education intervention in the Canadian primary care setting. The RISE Project has recently completed the qualitative strand of Phase 1 data collection; the quantitative strand for Phase 1 is currently ongoing. No results manuscripts have been generated or are under consideration.

The specific qualitative, quantitative, and mixed method research questions [33] informing each phase of the RISE Project are included in Table 1.

**Table 1:** RISE Project research questions

Phase of the RISE Project (timeline)	Methodological notation	Research questions
Phase 1 (2020–2021)	QUAL ➔ quan	*Qualitative research question:* How do trainee and licensed physicians and social workers residing in the provinces of Ontario, Quebec, and Alberta describe their learning and implementation needs and preferences related to education on family violence? *Quantitative research question:* Among the licensed and trainee physicians and social workers in Canada, what are the most salient learning and implementation needs and preferences related to education on family violence? *Mixed methods research question:* Are the learning and implementation needs and preferences related to education on family violence and which are described by a purposeful sample of trainee and licensed physicians and social workers in Ontario, Quebec, and Alberta confirmed by a broader population of physician and social work professionals in Canada?
Phase 2 (2021–2022)	((qual)QUAN)	*Qualitative research question:* How do trainee and licensed social workers and physicians in Ontario, Quebec, and Alberta articulate the usability of the VEGA intervention? *Quantitative research question:* What proportion of a sample of trainee and licensed social workers and physicians in Ontario, Quebec, and Alberta report adequate usability (including satisfaction, effectiveness, and efficiency) following the completion of the VEGA intervention? *Mixed method research question:* To what extent do the findings from the “think-aloud” protocols help to understand the usability scores generated from the quantitative strand of data collection?
Phase 3 (2022–2023)	QUAN + QUAL	*Quantitative research question:* Is it acceptable and feasible to implement and evaluate VEGA as a continuing education intervention to improve provider KASB related to recognizing and responding to family violence in Ontario, Alberta, and Quebec primary care settings? *Qualitative research question:* How do providers in primary care and their managers describe the acceptability and feasibility of VEGA as a continuing education intervention to improve their KASB for recognizing and responding to family violence? *Mixed method research question:* How does clinical consultation data, as well as interviews with providers and managers, help to describe the acceptability, feasibility, and perceived impact of implementing and evaluating VEGA as a continuing education intervention in primary care settings within Ontario, Quebec, and Alberta?

### Implementation and research framework

All phases of the RISE Project are informed by a novel application of the Active Implementation Frameworks (AIFs) [34–37]. The AIFs are a determinant framework that acknowledges the importance of considering multiple levels and types of influence in the uptake and sustainability of educational interventions [38]. The AIFs outline five key determinants of implementation and evaluation success, including (1) a usable innovation (i.e., educational intervention); (2) implementation stages; (3) implementation teams; (4) the identification and enactment of implementation drivers; and (5) the incorporation of improvement cycles [34–37, 39]. Previous work by members of our own team [40–45], as well as others [37, 46–48], indicates that implementation and evaluation efforts guided by the AIFs have resulted in effective practice change. With respect to implementation stages, it is important to note that stage 1 (exploration) is the point at which an organization (or stakeholder group) considers the need and fit of a usable innovation. This stage of the AIF was addressed in the preparatory work for the RISE Project via the engagement of “champions” representing eight national organizations of HSSPs, primarily physicians and social workers (see Table 2).

**Table 2:** Collaborating organizations

Organization	Approximate size of membership
Royal College of Physicians and Surgeons of Canada	52,000^a^
Canadian Psychiatric Association	2700
Canadian Association for Emergency Physicians	2500
Canadian Paediatric Society	3500
The Association of Faculties of Medicine	29,200^b^
College of Family Physicians of Canada	38,000
Child Welfare League of Canada	2000
Canadian Association of Social Workers	20,000

^a^It is important to note that HSSPs can hold multiple memberships across our collaborating organizations; thus, a particular physician (for example) could be counted in the approximation for the Royal College of Physicians and Surgeons of Canada as well as the Canadian Psychiatric Association. ^b^Approximate estimate includes undergraduate medical students, graduating medical doctors, and postgraduate trainees

## Methods

### Overall design

**Fig. 1:**
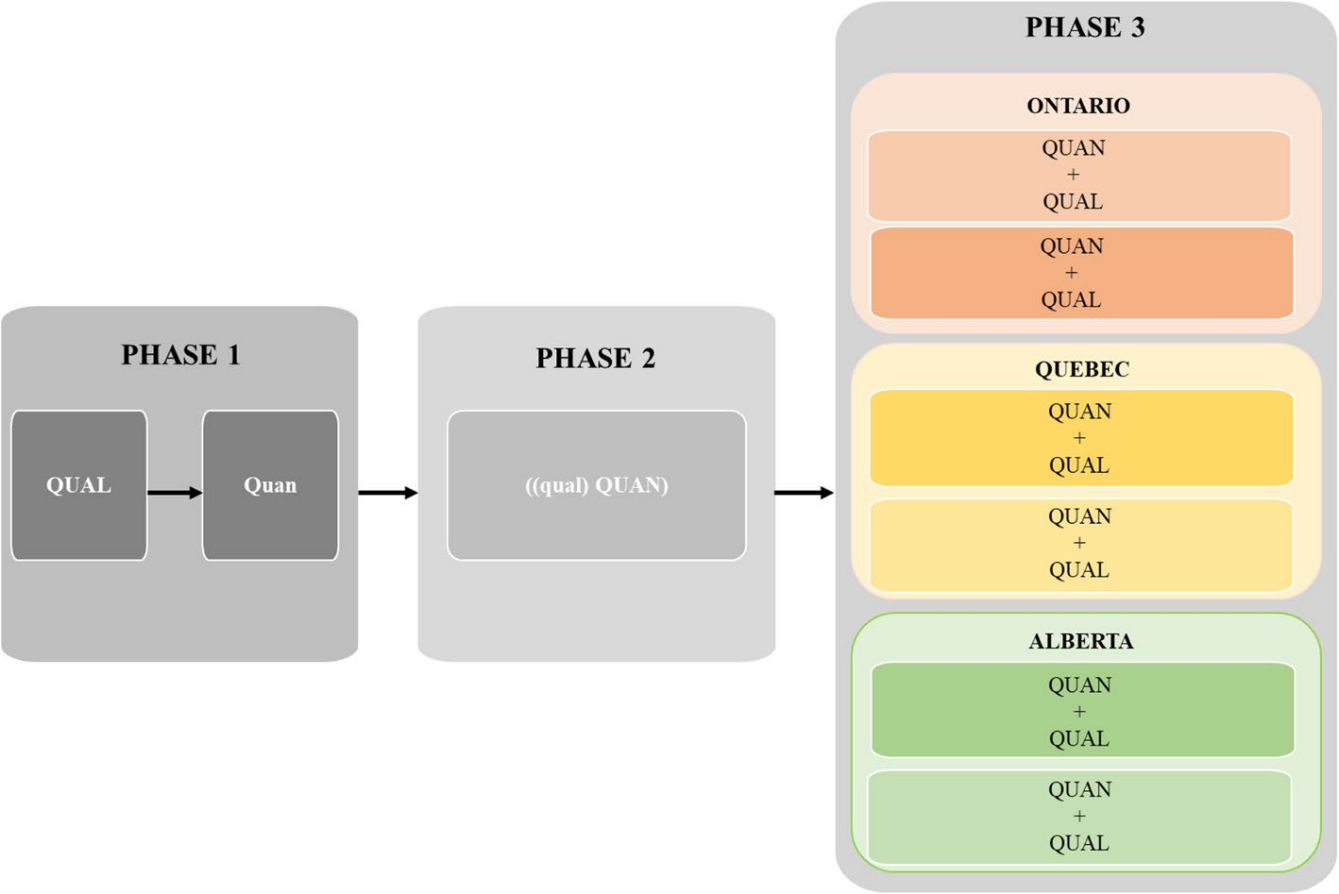
Overview of the RISE Project’s multiphase mixed method research design. The figure gives an overview of the RISE Project’s multiphase mixed method research design; it details three phases of research and each phase is characterized by its own mixed method research design. Phase 1 uses a sequential exploratory mixed method research design, which is given by the notation of QUAL ➔ quan. This notation indicates the qualitative strand occurs first, is given more weight, and informs the quantitative research strand. Phase 2 uses an embedded mixed method research design, which is given by the notation (qual(QUAN)). This notation indicates that although the phase starts with qualitative data collection, it is embedded within a larger quantitative paradigm; data for both strands are collected in the same data collection visit. Phase 3 uses a concurrent mixed method research design, which includes parallel collection of qualitative and quantitative data that occurs over multiple visits and analyzed separately (notation is QUAL + QUAN). The qualitative and quantitative data for each site in each province are weighted equally and findings for each strand, for each site, are integrated to create a comprehensive interpretation. The arrows connecting each phase indicate that some aspect of findings and methods (e.g., measures) from each phase, inform the next phase

To achieve its aims, the RISE Project is using an emergent, sequential, multiphase mixed method research design [33]. We are collecting and integrating qualitative and quantitative data over the course of three conceptually and methodologically linked research phases. Each of the three phases is driven by its own mixed method research design, with each phase given equal priority and purposefully connected via incorporating the findings of previous phases [33, 49]. Informed by the guidelines detailed by Creswell and Plano Clark [33], Fig. 1 provides a graphical overview of our multiphase mixed method research design. Details concerning the procedures for each phase are described below. The RISE Project has been approved by the Hamilton Integrated Research Ethics Board (Project #: 11295), the University of Calgary Conjoint Research Ethics Board (Project #: REB20-0338), and McGill University’s Research Ethics Board (Project #: 20-06-038). The Standards for Reporting Framework for Implementation Studies (STaRI) Checklist has guided the reporting of our protocol [1]. Our completed checklist can be found in Supplementary File 1.

### Setting and participants

The majority of the RISE Project activities will take place in the Canadian provinces of Alberta, Ontario, and Quebec. Up to one-third of adult Canadians have reported exposure to child maltreatment or intimate partner violence in their lifetime [50, 51] and rates of police-reported family violence constitutes 26% of all police-reported crime in the nation [52]. Our focus on the social work and medical disciplines, as well as the provinces of Alberta, Ontario, and Quebec, were justified on the bases of five key reasons: (a) social workers and physicians are among the top three largest groups of health care and social service providers in Canada; (b) Alberta, Ontario, and Quebec are among the most populous of the Canadian provinces and contain a high proportion of the HSSPs with membership to our collaborating organizations; (c) the self-reported prevalence rates of family violence in each these three provinces are greater than 25%; (d) each province’s selection represents the range in the proportion of provincial residents having the ability to speak both of Canada’s official languages, English and French (Quebec = high proportion of the population is bilingual (44.5%); Ontario = moderate proportion (11.2%); Alberta = low proportion (6.6%)); and (e) each province has created primary care teams that are inclusive of a range of HSSPs that are likely to come into contact with individuals exposed to family violence, which is of particular relevance for phase 3 of our work [53–60].

### Phase 1: The scope and salience of learning and implementation needs and preferences — a sequential exploratory mixed method study

Guided by stage 2 (installation) of the AIF, phase 1 of the RISE Project will determine the scope and saliency of the learning and implementation needs and preferences of social workers and physicians related to continuing education in family violence. According to Metz et al. [36], the installation phase of a new intervention is an often overlooked, but necessary stage for implementation success. During this stage, identifying factors for optimizing intervention delivery, uptake, sustainability, and impact (e.g., policy and practice drivers) is the focus. The objectives for phase 1 will be addressed using a sequential exploratory mixed method design [33]; qualitative and quantitative data will be collected sequentially from trainee and licensed physicians and social workers who are training or practicing in each of the three provinces of focus. The term “exploratory” denotes that this phase of the project will be qualitatively dominant, with the measures for the quantitative strand influenced by the initial qualitative findings [61].

#### Qualitative research strand

##### Design, sampling, and recruitment

Our team has drawn on the principles of qualitative description to guide sampling, data collection, and analysis procedures for the qualitative strand of phase 1 [62, 63]. Criterion-based sampling strategies will be used to recruit approximately 100 participants for this strand of data collection [64, 65]. We will operationalize criterion-based sampling via predefined eligibility criteria to recruit participants who (a) are 18 years of age or older; (b) are an undergraduate or graduate-level medical or social work trainee (with at least one clinical placement/practicum in the last 12 months) or practicing physician or social worker; and (c) reside in the province of Quebec, Alberta, or Ontario. Recruitment of participants will occur via a three-pronged approach, including the distribution of recruitment e-mails via our collaborating organizations. This process will be supplemented with the posting of recruitment materials on social media platforms, as well as e-mail requests for participation circulated to the professional networks of the study team. Third, snowball sampling methods will be used with each “source” participant to accumulate additional participants over time [65].

##### Qualitative data collection and analysis

Enrolled participants will be asked to complete a one-on-one, semi-structured qualitative interview with a member of the research team by Zoom or telephone [66]. A semi-structured interview guide consisting of 5–7 key, open-ended questions will guide data collection and are informed by the research objectives for phase 1. In keeping with the traditions of qualitative description, interview questions will be adapted throughout data collection to explore patterns in the data. Interviews will last between 30 and 45 min and will be audio-recorded and transcribed verbatim by a professional transcription service working with the research team. Demographic data will be collected from each participant using a short demographic questionnaire. Each participant will receive a $75.00 honorarium in the form of an e-gift card as a token of appreciation for their completion of the interview.

This phase will use an inductive and deductive approach to analyze participant descriptions of their needs and preferences related to education on family violence to produce: (a) new practice-based insights about the type and extent of family violence education that is needed among our disciplinary groups and (b) a detailed summary of drivers needed to scale-up and sustain family violence education, and VEGA more specifically, among HSSPs in Canada. Inductive conventional content analysis [67] of interview transcripts using the constant-comparison technique will identify pertinent concepts and constructs related to participants’ perceived learning and implementation needs and preferences related to education in family violence; it will also allow for an examination of the extent to which needs and preferences are consistent versus distinct across disciplines and field of specialties (e.g., community mental health; emergency medicine; etc.,). Summative content analysis, which is a deductive analytical technique [67], will provide counts of needs and preferences that are identified by participants; this information will aid in the interpretation of the results by demonstrating the learning and implementation needs and preferences that are most relevant for physicians versus social workers, as well as those that may be more or less salient across each discipline and field of specialty [68, 69].

#### Quantitative research strand

##### Design, sampling, and recruitment

The quantitative strand will corroborate and extend the findings from the qualitative strand of phase 1 and determine the frequency of family violence education learning and implementation needs and preferences within and across groups of Canadian providers and trainees at a national level; this will allow our team to make empirically supported decisions related to strategies for supporting the scalability and sustainability of family violence education — and VEGA more specifically, should it prove to be an effective educational intervention. The quantitative strand will follow a quantitative, cross-sectional survey research design [70]; the primary sampling frame will be the membership registries of our collaborating organizations, which provide lists of members who have provided consent to the respective organization to have their e-mail address available for research purposes. Using non-probability, opportunity-based sampling, eligible participants will be those who (a) are 18 years of age or older, (b) are a trainee or practicing physician or social worker residing in any Canadian province or territory, and (c) can provide informed consent and complete the self-report survey in either English for French. A request will be made to the administrator of each collaborating organization to distribute our recruitment and data collection materials via e-mail to individuals on the research registries and which directs interested participants on how to complete research activities. This process will be supplemented with the posting of recruitment materials on social media platforms, as well as e-mail requests for participation circulated to the professional networks of the study team.

##### Quantitative data collection and analysis

Informed consent and data collection procedures will be completed anonymously via Lime Survey at the participant’s convenience during a 4-week study window. Survey items will be informed by the coding categories and constructs identified in the qualitative strand of phase 1 (described above), as well as include validated measures from health professions education, implementation science, and family violence literature. Specifically, items will ask participants to self-report on their (a) socio-demographic characteristics and previous training in family violence; (b) readiness to undertake family violence training (e.g., Brief Readings to Change Scale [71]); (c) attitudes toward incorporating research evidence into their practice (e.g., Evidence Based Practice Attitudes Scale); (d) preparedness to address family violence in practice (e.g., an adapted Physician Readiness to Manage Intimate Partner Violence Survey (PREMIS) [72, 73] and Healthcare Provider Attitudes toward Child Maltreatment Reporting Scale [74, 75]); and (e) preferences, barriers, and facilitators related to participating in education activities focused on family violence (e.g., online vs. face-to-face learning, etc.) [76]. Participants who complete the anonymous survey will be given the option to enter their name in a draw to win one of six $500.00 honorariums.

All data from our Lime Survey platform will be exported into our data analysis software, SPSS (version 28). Estimates of the variability and saliency of HSSP learning and implementation needs, and preferences will be generated via statistics of dispersion and central tendency. Differences across provider groups and specialities will be evaluated using single-level correlation and regression analysis.

### Phase 2: VEGA usability and exploratory assessment of education outcomes

According to the AIFs, stage 3 (initial implementation) refers to the point at which changes begin to occur within the overall practice environment. For the purposes of this project, this would include not only changes in HSSP KASB, but also changes in overall health and social service sector capacity to be able to safely recognize and respond to family violence. Guidelines from the developers of the AIF indicate that stage 3 is the point at which implementation challenges present themselves, as do opportunities to refine and expand the suite of strategies to support successful implementation and realize intervention impact [36]. Given the probability of implementation failure to occur at this stage, data-driven, pilot-based approaches are encouraged [77, 78]. For this reason, we will pilot VEGA through the application of usability testing. Usability testing focuses on the evaluation of intervention technology and is an essential step for realizing e-learning impacts; this is especially the case in health professions education [79–82].

Usability testing follows a data transformation variant of an embedded mixed method research design to generate a description of (a) intervention usability (i.e., the extent to which VEGA can be used by HSSP users to achieve their learning goals with effectiveness, efficiency, and satisfaction) and (b) educational impact [83]. Using this design, quantitative and qualitative strands of data collection are collected from the same participants during one data collection visit.

#### The intervention: Violence, Evidence, Guidance, Action (VEGA) project

VEGA is an online educational intervention that was created for Canadian HSSPs to develop and improve their KASB related to recognizing and responding to all forms of family violence. The intervention was developed using an iterative design process that incorporated systematic evidence reviews of the family violence literature, environmental scans of existing and relevant training resources, and consultation with clinical and research experts in the areas of family violence, instructional design, and health professions education. An important element of VEGA’s development also included repeat consultation from clinicians and scientists belonging to 22 national-level HSSP organizations in Canada.

VEGA follows a participatory, encounter-based curriculum over the course of four core learning modules; learning module content and pedagogical approaches are informed by evidence-based models of adult learning and cognitive processing [84–88] and follow the VEGA Competency Framework for Recognizing & Responding Safely to Family Violence. VEGA can be completed as a self-directed educational activity or it can be delivered by trained facilitators in a virtual or face-to-face workshop. Time to completion is approximately 3 h. Although freely available to HSSPs across Canada, the intervention has yet to undergo formal evaluation to determine its effectiveness. More information about VEGA and its competency framework can be found at vegaproject.mcmaster.ca.

#### Qualitative research strand

##### Sampling and qualitative data collection

Evidence indicates that 95% of usability problems can be identified with usability testing among a purposeful sample of five-to-ten potential end-users. In the case where the intent of the intervention is to meet the learning needs of several different end-users, there is a need to ensure there is sufficient user-testing with each user type [89, 90]. Given this information, we will use purposeful, criterion-based sampling [64, 65] to recruit a convenience sample of up to 20 trainee (~ 10 social work; 10 physician) and 20 licensed practitioners (~10 social work; ~ 10 practitioners) to participate in this phase of the project. Eligible participants will be those who are (a) 18 years of age or older; (b) a trainee or practicing physician or social worker residing in Hamilton, Calgary, or Montreal; and (c) willing to complete user-testing procedures synchronously with a member of the research team.

Enrolled participants will complete the self-directed format of the VEGA intervention using a think-aloud protocol [89]. Specifically, the participant will be prompted by a member of the research team to navigate through the VEGA intervention to complete a series of learning tasks that follow VEGA’s recommended learning pathway while “thinking aloud.” That is, participants will be prompted by the research team member to say their thoughts and activities “out loud” as they move through the intervention (e.g., “I am now scrolling down to see more content;” “I can’t locate the button to move forward in this module”). The think-aloud protocol will be audio-recorded and transcribed verbatim for qualitative data analysis. All participants will be offered continuing medical education credits (physicians; physician trainees) or a certificate of continuing education participation (social workers; social work trainees) for completing the VEGA intervention via the think-aloud protocol.

##### Qualitative data analysis and data transformation

De-identified think-aloud transcripts will be analyzed using framework analysis [91, 92]. This process involves the a priori indexing of the types of usability problems detailed by Hornbaek [93] and Hvannberg and Law [94] into NVivo data management software and completing iterative reviews of the transcripts to apply the indexed usability problems to the transcribed data. Application of usability problems to the data will be completed independently by two members of the research team and will allow for the determination of usability problems (if any), problem types, their frequency, and their severity (1 = mild problem, 2 = moderate problem, 3 = serious problem, 4 = critical problem). Differences in indexing of user problems will be resolved via consensus discussion among the analysts and the leads of the research team.

#### Quantitative research strand

##### Data collection

The quantitative strand of data collection follows a pre-post-research design [95]. Specifically, the sample participants who enroll in the qualitative portion above will self-complete a series of research assessments on our Lime Survey platform at two timepoints: immediately prior (time 1; pre-assessment) and immediately following their think-aloud protocol (time 2; post-assessment). Research assessments will be embedded in a hyperlink that is sent to participants via email 25 min prior to the start of the think-aloud protocol, as well as again immediately after the think-aloud protocol. In addition to socio-demographic characteristics (sex at birth, gender identification, age, location, professional status and discipline, years of practice), quantitative assessments pre- and post-the think-aloud protocol will include the same validated measures of KASB administered in the quantitative strand of phase 1, as well as a vignette-based assessment of knowledge and skill accuracy related to recognizing and responding to family violence. Vignette-based assessment methods are a common, robust measure of practitioner knowledge and skill accuracy related to family violence [30–32], as well as in medical and health professions education, more generally [96–98].

The post-assessment will also ask participants to self-report the extent to which they perceive their current clinical environment to be safe to discuss complex issues related to family violence (e.g., Safety Culture Scale [99, 100]), as well as their satisfaction with usability of the VEGA intervention (e.g., System Usability Scale (SUS)) [101, 102]. Validated measures will be supplemented with data that is compiled and tracked by the project’s research staff; this will include tracking (a) VEGA usability effectiveness via “learning task completion” during the think-aloud protocol with a yes/no checklist, (b) user time on “learning task,” (c) missing data at the item and group level at the pre- and post-assessment timepoints, and (d) the average time needed to complete pre- and post-research assessments. An honorarium ($150.00 for a practicing social worker or physician; $75.00 for resident physicians and trainee social workers) in the form of an e-gift card will be provided as a token of appreciation for the completion of quantitative measures.

##### Quantitative and integrated data analysis

Quantitative data will be analyzed and interpreted via estimates of central tendency and dispersion. Specifically, the proportion of participants reporting “satisfactory” usability for the VEGA intervention (i.e., a score of > 70) will be generated for trainees and practitioners by discipline (social work, medicine) [89, 93, 94]. In addition, the mean and range of SUS scores for the entire sample will be reported, as will the mean and range for participants who reported an SUS adjective rating of (a) poor, (b) “OK,” (c) good, (d) excellent, and (e) best imaginable. We will compute the range of missing data for all quantitative measures, with feasibility of collecting quantitative outcome data indicated by less than 20% missing data at the participant and group level for each timepoint. We will also generate and present (a) correlations between continuous user satisfaction scores and pre/post-practitioner scores on KASB measures and (b) cross-tabulations of satisfaction scores, the rate of usability problems, problem types, and problem severity [33, 103]. Upon review of results, team members will decide which usability changes — if any — need to occur prior to initiating phase 3 of the RISE Project. We will partner with the VEGA team to implement those changes to the intervention.

### Phase 3: Determining the acceptability and feasibility of implementing and evaluating VEGA in primary care

Continuing to be guided by stage 3 (initial implementation) of the AIF, the primary aim of phase 3 is to determine the acceptability and feasibility of implementing and evaluating VEGA to improve HSSP KASB related to recognizing and responding to family violence in the primary care setting. Our secondary objectives for phase 3 are to determine exploratory estimates of the educational impact for the VEGA intervention, as well as describe the usefulness of implementation strategies to support and sustain VEGA educational impacts in the primary care setting. We will address our objectives using a concurrent mixed method research design; quantitative and qualitative strands of data collection and analysis will occur in parallel and be given equal priority [49]. Quantitative data will provide essential information about HSSP enrollment, retention, attrition, and data completeness; we will also generate exploratory estimates of education effect and variance. The qualitative strand of data collection will provide corroborating information about the acceptability and feasibility of the VEGA intervention in the primary care setting, as well as HSSP perceptions of perceived value and impact of the VEGA intervention and our implementation strategies. Table 3 details the specific acceptability, feasibility, and proof-of-concept objectives for phase 3 mapped to their type of outcome assessment, any relevant hypotheses, and analysis.

**Table 3:** Primary and secondary objectives, outcome variables, hypotheses, and analysis for phase 3 of the RISE Project

Objective	Focus	Outcome measure; ***data source***	Criteria for success/hypothesis	Analysis
**Primary objective “A”**				
Determine the proportion of primary care clinics who are approached and agree to participate	Feasibility of clinic recruitment and retention	(i) Percentage of eligible clinics who are approached and enroll; *RC tracking*	**Criteria for success** (i) A 60% or greater enrollment rate of primary care clinics approached for participation	(i) Descriptive counts, percentages, and range across provinces
**Primary objective “B”**				
Determine the proportion of primary care providers who (i) enroll in the study, (ii) complete all VEGA modules, (iii) are retained for all follow-up timepoints	Acceptability and feasibility of VEGA education Acceptability and feasibility of staff recruitment and retention procedures	(i) Percentage of eligible providers that enroll; *RC tracking* (ii) Percentage of enrolled providers that complete all VEGA modules; *RC tracking* (iii) Percentage of providers that complete quantitative research assessments at all timepoints; *RC tracking*	**Criteria for success** (i) A 60% or greater enrollment rate of providers at each of the clinics; (ii) A 70% or greater VEGA completion rate among enrolled providers; (iii) 70% or more of enrolled providers will be retained at a 12-week follow-up	(i–iv) Descriptive counts, percentage, and range across provinces
**Primary objective “C”**				
Determine the feasibility of collecting provider-level outcome data at baseline, 1-, and 3-month follow-up timepoints	Feasibility of secondary outcome data collection	Percentage of missing data on secondary outcomes at each time point; *RC tracking* Percentage of missing research assessments; *RC tracking*	**Criteria for success**: There will be less than 20% missing data at the individual and group level for each timepoint Our RC is able to generate estimates of effect and variability (with 95% confidence intervals) for the secondary outcomes	Percentages and range of missing data at the item, individual, and group level across sites for each of the secondary outcome measures and across secondary outcome measures Regression estimates and associated confidence intervals
**Primary objective “D”**				
Explore the acceptability and feasibility of the intervention and evaluation procedures in (i) a sub-sample of providers who fully completed the VEGA intervention, as well as (ii) managers of participating primary care clinics	Acceptability of training Acceptability of research procedures Proof-of-concept/clinical impact	Narrative descriptions of perceived acceptability and burden of intervention and research activities; *qualitative interviews with providers and clinic managers* Narrative description of perceived value and impacts of intervention in practice; *qualitative interviews with providers and clinic managers.* Narrative descriptions of implementation successes and challenges; *consultation sessions with providers*	**N/A**	Directed content analysis; summative content analysis; thematic analysis
**Secondary objective (A)**				
Describe the change in provider (i) knowledge and skill accuracy to recognize and respond to IPV and child maltreatment, as well as change in their perceived; (ii) preparedness to recognize and respond to IPV and child maltreatment; (iii) attitudes towards recognizing and responding to IPV and child maltreatment from baseline to post-intervention and 3-month, follow-up timepoints	Proof of concept/educational impact	Quantitative research assessments; *provider self-report*	**Hypothesis:** Self-reported knowledge and skill accuracy (*Child Maltreatment Vignette Scale; Actual Knowledge Subscale - PREMIS*), preparedness (*Mandatory Reporting Self-Efficacy Scale; Preparedness Subscale-PREMIS measure*), attitudes (*Role Beliefs Scale*) will be better at all follow-up timepoints, relative to baseline	Regression analysis
**Secondary objective “B”**				
Describe the change in the number of referrals made by enrolled providers over the previous month to (i) intimate partner violence services; (ii) parenting services/interventions; (iii) child welfare services; or (vi) psychotherapy services at baseline, post-intervention, and 3-month follow-up timepoints	Proof of concept/educational impact	Quantitative research assessments; *provider self-report*	**Hypothesis:** Provider self-reported referrals of patients to (i) intimate partner violence services; (ii) parenting services/interventions; (iii) child welfare services; or (vi) will be higher at all follow-up timepoints, relative to baseline	Regression analysis

#### Quantitative research strand

##### Design, sampling, and recruitment

The quantitative strand will follow a non-experimental, repeated measures design [104]. HSSPs working in primary care clinics in the provinces of Ontario, Alberta, and Quebec will be recruited to undergo the VEGA intervention and complete quantitative measures of education outcomes at multiple timepoints. Measures will be administered to determine (a) the acceptability and feasibility of collecting data on proposed educational outcome measures for a definitive trial and (b) generate preliminary estimates of effect (i.e., proof-of-concept) and variability, which can inform sample size estimations for a definitive trial. To achieve our research aims, we will use a three-stage sampling strategy; two of the three stages are relevant for the quantitative strand of data collection.

Stage 1 will occur at the clinic level. Criterion and simple random sampling strategies will be used to enlist two primary care clinics in the provinces of Ontario, Quebec, and Alberta for participation [64, 65]. Given that samples of approximately 40 participants are generally sufficient for acceptability and feasibility studies [105–107], a roster of primary care clinics with a front-line complement of between 10 and 60 HSSPs who provide health and/or social services to individuals and families in each of the eligible provinces will be generated via provincial registries made publicly available on each province’s Ministry of Health website. Two clinics per province will be selected from these rosters for participation via a simple random sampling algorithm in SPSS. Directors of the selected clinics will be contacted by the research team via email and provided with a one-page overview of the project and the opportunity to meet synchronously with the research team to address study-related questions. Directors will be asked to provide consent for clinic participation, as well as the email addresses for all HSSPs within the clinic. To be eligible to participate, the Director of each of the selected clinics must (a) provide permission for the full complement of their HSSP staff to complete study-related activities and (b) have no ongoing participation in other education-related research projects. Random selection of replacement clinics will continue until we achieve our clinic sample aims.

Stage 2 sampling will occur at the individual level; full-population sampling will involve inviting all full-time and part-time HSSPs at each of the enrolled clinics to participate in the quantitative strand of our study [108]. We anticipate that this individual, convenience sampling approach will yield between 5 and 20 practitioners participating at each of the respective clinics. Eligible practitioners will be those who (a) have worked for the selected clinic for a minimum of 2 months, (b) intend on working (to the best of their knowledge) at the clinic until the end of the study, and (c) provide informed consent and are willing to complete quantitative and qualitative study procedures.

#### Implementation and educational intervention

##### Preparatory clinic webinars

Preparatory webinars with each of the enrolled clinics and their participating HSSPs will be conducted prior to launching baseline data collection (detailed below). Two members of the research team will facilitate the webinars with the purpose of increasing engagement in project activities, addressing any process queries prior to the launch of the data collection, and supporting readiness for implementation and evaluation activities [109–111].

##### Educational intervention

Enrolled HSSPs at each of the participating clinics will undergo a course of self-directed VEGA or workshop VEGA, as outlined in phase 2. This phase will be influenced by the perspectives of our collaborating organizations, the health professions education literature, as well as findings of phase 1 and phase 2 of the RISE Project. Should a clinic pursue the self-directed option of the VEGA intervention, each participant within the clinic will be provided with VEGA login and password information, as well as instructions to complete all VEGA learning modules within a 4-week period (i.e., the intervention period). Reminders for intervention completion will be automatically generated and sent to participants every week via the Lime Survey interface until intervention completion or until the participant’s intervention period has passed. Should the workshop format of VEGA be selected, all HSSPs in each clinic will be invited to participate in a VEGA workshop (one per clinic), co-facilitated by at least two trained facilitators from the VEGA team. Following the completion of intervention and data collection activities, workshop participants will be provided the log-in information to have unrestricted access to the self-directed format of the VEGA modules.

##### Participant consultation

Tri-weekly participant consultation will be provided via Zoom by two clinician members of the research team following each clinic’s intervention period. Consultation will focus on supporting HSSP clinical application of family violence recognition and response principles detailed in the VEGA curriculum [110, 112]. Consultation sessions will be 45 min in duration, audio-recorded, and transcribed verbatim for qualitative data analysis (detailed below).

##### Quantitative data collection

Quantitative data related to acceptability and feasibility will be collected by the project’s research coordinator (RC); this will include tracking the number of clinics approached for participation, who request preparatory webinars, and who enroll. It will also involve tracking HSSPs within each clinic who (a) inquire about participation, (b) are eligible, and (c) enroll. The RC will also record the number of (d) (i) contacts needed to complete consent procedures and (ii) contacts to arrange all research assessments and the number of HSSPs that (e) complete VEGA training modules; (f) dropout following consent; (g) could not be reached for follow-up; (h) complete quantitative research assessments at each timepoint; and (i) are approached, agree, complete, and withdraw from the qualitative data collection strand.

Quantitative data regarding VEGA educational outcomes will be collected via HSSPs’ self-completion of assessments administered by email at three timepoints: 1 week before the intervention period (time 1; baseline), immediately following the completion of the intervention/intervention time period (time 2; post-intervention), and 3 months following intervention completion (or the intervention period for participants who take the full-time frame or who do not complete all of the VEGA learning modules) (time 3; 3-month follow-up). Given that this work is based within the overall emergent, multiphase, mixed method research design, we anticipate that measures capturing education outcomes will be the same as those administered in phase 2 of the RISE Project, which includes a brief assessment of socio-demographic characteristics. However, it is possible that these measures may expand or change throughout the duration of the research program. Additionally, at each timepoint, we will ask participants to report on the number of referrals made over the previous month to (i) intimate partner violence services, (ii) parenting services/interventions, (iii) child welfare services, or (vi) psychotherapy services.

##### Quantitative data analysis

Given our primary focus on acceptability and feasibility, quantitative data for our primary objectives in phase 3 will be analyzed using descriptive statistics. In addition, based on our integration of sampling and recruitment recommendations in the literature [2, 3], we have proposed a priori thresholds for acceptability and feasibility as follows: the proportion of (a) primary care clinics and their HSSPs agreeing to participate will be 60% or greater; (b) enrolled HSSPs who complete all modules of the VEGA intervention will be 70% or greater; (c) missing data for each timepoint will be less than 20% at the HSSP and clinic levels; and (d) our team will be able to generate estimates of effect and variability for education outcome measures. Informed by the AIF and the broader implementation science literature, if more than one of these thresholds are not met, our team will consider revisions to our implementation and research procedures before proceeding to a definitive trial. Given our use of mixed methods, we expect that our qualitative data (outlined below) will be especially helpful for understanding how and why thresholds were or were not met and what can or should be augmented in the implementation and research procedures to bolster the possibility of future evaluation success. Secondary objectives will be addressed using regression analysis, with results being reported as estimates of effect (95% confidence interval) and associated *p*-values. Analyses will be exploratory, with no adjustments for multiple comparisons.

#### Qualitative research strand

##### Design, sampling, and recruitment

The qualitative strand of phase 3 will follow the principles of qualitative description [62, 63]. Driven by eligibility and enrollment procedures for the quantitative strand of data collection, we will use criterion-based sampling to select a sub-sample of HSSPs (*n* = 5–10, per clinic) who provided quantitative data to participate in a qualitative semi-structured interview. This sub-sample of HSSPs will be asked to complete an interview at two timepoints: (a) within 1 week of intervention completion and (b) 2 weeks following their submission of the 3-month quantitative research assessment. We will invite HSSPs who represent various genders, education levels, employment tenure, and previous training in family violence to participate in this strand of data collection. We will also recruit up to three managers, directors, or administrators (i.e., “managers/management”) from each of the enrolled clinics to participate in this strand of data collection. Management interviews will begin immediately following the intervention period for enrolled HSSPs in the same clinic. Eligibility and consent of HSSPs will have been obtained during quantitative study enrollment; consent for qualitative data collection will be verbally reconfirmed by the RC prior to qualitative data collection. Eligible managers will be those who have been working in their management role at the enrolled clinic for at least 6 months and who intend on working at the same clinic for the duration of the study.

##### Qualitative data collection

One-on-one interviews are a recommended method of data collection in applied qualitative research of interventions; they are a flexible approach that enables the gathering of an in-depth, first-hand account of a phenomenon in its given context [113]. A semi-structured interview guide consisting of 5–7 key, open-ended questions will guide data collection at each of the clinics; the guide will focus on the participants’ perception of the educational intervention and perceived impact, as well as the acceptability and burden of research, educational, and implementation support activities. HSSP and management interviews will be scheduled for between 45 and 60 min via Zoom at a time that is convenient for participants. Zoom is an externally hosted cloud-based service provided to the research team through our university. A link to Zoom’s privacy policy (https://zoom.us/privacy#_Toc44414835) will be provided to all potential participants in the consent form. Our research team will take all available precautions to reduce the risk of a privacy breach, including generating a unique Zoom link for each interview and providing a unique password for entry to each interview. In addition, each individual participant will be asked to refrain from using the video feature of Zoom, so that only the verbal content of each interview is audio-recorded for verbatim transcription. Management participants will also be asked to complete a brief, demographic information form. Qualitative interview participants will be provided a $75.00 honorarium in the form of an e-gift card at the completion of the interview.

##### Qualitative and integrated analyses

Qualitative data collection and analysis for each clinic will happen concurrently and begin immediately following the clinic’s intervention period; remaining clinical consultations and semi-structured interviews within and across clinics will have the opportunity to be informed by interim analysis of transcripts of both data types. Analysis of interview and clinical consultation transcripts will involve conventional and summative content analysis [67] to generate “within clinic” and “cross-clinic” summaries of acceptability, feasibility, and impact. Our data report will also incorporate counts of any perceived barriers and facilitators to acceptability and feasibility (alongside excerpts of qualitative data) identified in the interview or clinical consultation data via a mixed methods joint display. In addition, a modified stem-and-leaf plot will cross-tabulate scores on VEGA education outcome measures with qualitative excepts describing the perceived value and impact of the VEGA intervention and our implementation supports [114].

##### Rigor and integration across research phases

Strategies for ensuring the quality of mixed methods research continue to emerge in the methodological literature; the specific strategies for ensuring the rigor of multiphase mixed method research designs remain unclear. There is consensus however that rigor in any mixed methods research study requires prerequisite consideration of the expectations for rigor in the qualitative and quantitative strands of data collection. Informed by this, as well as guidelines articulated by Kefting [115] and O’Cathain [116], a range of strategies will be applied within and across all phases of our research program to achieve credible (internally valid), dependable (reliable), applicable (transferable, externally valid), and confirmable (neutral) findings [115, 116]. This includes (a) the commitment to publish the results for each phase of the RISE Project according to the Good Reporting of Mixed Methods Study Guidelines [116] as well as guidelines relevant to each phase of our work; for example, the Standards for Reporting Framework for Implementation Studies [117] and the CONSORT extension to pilot trials [118, 119] (both relevant for phase 3); (b) the purposeful integration of qualitative and quantitative approaches at the design, sampling, data collection, data analysis, or interpretation stages for each of the three phases of work; (c) employing psychometrically validated quantitative research instruments and repeat measurement approaches for phase 2 and phase 3; (c) a detailed audit trail throughout the entire research program; (d) field notes and analytical memos during qualitative data collection and analysis; and (e) investigator, data source, and data method triangulation where possible and appropriate [120, 121]. A detailed description for the strategies to ensure methodological rigor for each phase of the RISE Project will be reported in each phase’s result publication, respectively.

## Discussion

The key factors for successful uptake and sustainability of evidence-based educational interventions for HSSPs remain speculative. Recent work by Thomas [122], Price [123], and Carney [124] speak to the potential for models of implementation science to reduce the chasm between the development, implementation, and sustainability of educational interventions among the health and social service professions. Critically, our model of implementation science acknowledges that interventions to improve HSSP KASB related to family violence — including the uptake, impact, and sustainability of VEGA — take place within a complex context of macro (e.g., regulatory college support, accreditation), meso (e.g., institutional policies and support), and micro (e.g., provider) factors; each of these factors needs to be identified and considered in implementation efforts given their potential role in influencing intervention take-up and therefore the ability to actualize the intervention primary (i.e., educational) and secondary (i.e., health) outcomes [111, 125–128].

The present study has several strengths. First is its commitment to identify and operationalize the key drivers of family violence education uptake (and VEGA uptake more specifically) and sustainability throughout the remaining phases of the work and over the long-term, which is likely to yield more rapid translation of education and health outcomes attributable to educational interventions. In addition, the triangulation of data types from trainees and practitioners in three provinces and from two of Canada’s largest HSSP disciplines [53] will enhance the credibility, transferability, and trustworthiness of our findings; it will also generate a more comprehensive understanding of family violence education drivers, perceived value and impact, and acceptability and feasibility for the evaluation of VEGA in the real world. A third and critical strength of the RISE Project is a strong collaboration with eight national-level HSSP organizations who are key advocacy bodies for continuing health professions education among social workers and physicians in Canada.

There are three principal limitations of the RISE Project. The first is the exclusion of other disciplines in two of the three phases of work. Second, the quantitative strands of data collection in phase 2 and phase 3 follow non-randomized designs, which precludes the possibility of making causal claims of intervention impact. Third, none of the phases will involve the collection of data from the perspective of individuals exposed to family violence. We have designed a separate study to elicit the perspectives of those who have survived family violence or who work as community advocates for survivors, and we expect to integrate the learning from that project into the present work, as project activities progress.

The RISE Project was developed with the overall objective of initiating a robust evidence-base concerning the need and preferences related to family violence education among HSSPs in Canada, as well as generate initial information about the value and impact of VEGA to improve HSSP recognition and response to family violence in their practice encounters. Informed by a model of implementation science, the AIFs, the research program has a central emphasis on identifying and addressing key drivers of family violence education uptake, sustainability, and impact among the HSSPs throughout project activities. This model provides a constructive framework for considering how the broader impacts of VEGA — or any other family violence educational intervention — can be quantified, explained, and leveraged within and across HSSPs and their service contexts, more generally.

## Supplementary Information

**Additional file 1.** Standards for Reporting Implementation Studies: Completed StaRI Checklist.

## Data Availability

Not applicable.

## Abbreviations

AIF: Active Implementation Frameworks
HSSP: Health and social service provider
KASB: Knowledge, Attitudes, Skills, and Behaviors
RISE Project: Researching the Impact of Service provider Education Project
VEGA: Violence, Evidence, Guidance, Action
